# A review of neuroimaging studies of race-related prejudice: does amygdala response reflect threat?

**DOI:** 10.3389/fnhum.2014.00179

**Published:** 2014-03-27

**Authors:** Adam M. Chekroud, Jim A. C. Everett, Holly Bridge, Miles Hewstone

**Affiliations:** ^1^Department of Experimental Psychology, University of OxfordOxford, UK; ^2^Oxford Centre for Functional Magnetic Resonance Imaging of the Brain (FMRIB), John Radcliffe Hospital, Oxford UniversityOxford, UK

**Keywords:** amygdala, prejudice, neuroimaging, social neuroscience, implicit bias, threat

## Abstract

Prejudice is an enduring and pervasive aspect of human cognition. An emergent trend in modern psychology has focused on understanding how cognition is linked to neural function, leading researchers to investigate the neural correlates of prejudice. Research in this area using racial group memberships has quickly highlighted the amygdala as a neural structure of importance. In this article, we offer a critical review of social neuroscientific studies of the amygdala in race-related prejudice. Rather than the dominant interpretation that amygdala activity reflects a racial or outgroup bias *per se*, we argue that the observed pattern of sensitivity in this literature is best considered in terms of potential threat. More specifically, we argue that negative culturally-learned associations between black males and potential threat better explain the observed pattern of amygdala activity. Finally, we consider future directions for the field and offer specific experiments and predictions to directly address unanswered questions.

## Introduction

An emergent trend in modern psychology, spurred by the ability to non-invasively image the human brain and its neural activity, has focused on understanding how cognition is linked to neural function. As an enduring and pervasive aspect of human cognition, researchers have recently investigated the neural correlates of prejudice, broadly defined as any state of mind, feeling, or behavior that criticizes or derides others on account of a social group to which they may belong (Allport, 1954; Brown, 2010). Prejudice has been a core component of many of the defining events of the last century. Prejudice directed at different ethnic or religious groups has driven severe conflicts across the globe, from Nazi Germany to the former Yugoslavia to Rwanda. These extreme examples aside, there are other—and more pervasive—forms of prejudice: based on gender, age, sexuality, class, religion, or (dis)ability. While there exist many common forms of prejudice, the largest number of studies exist for race-related prejudice. It is, indeed, substantially more difficult to directly address other group memberships not outwardly expressed, such as religion or sexuality, in the same manner. Research into race-related prejudice quickly highlighted the amygdala as a brain region of interest (Hart et al., 2000; Phelps et al., 2000), although little is currently known of how responses differ across the structural and functional subdivisions of this structure.

Although effects of race on neural activity can be small, the behavioral impacts can be large, with repercussions for economic, legal, and medical decisions (for an excellent review see Kubota et al., 2012). Experimentally, one recent study found that differential brain activity for black and white faces predicts damage awards in hypothetical employment discrimination cases (Korn et al., 2012).

In combining the social psychological backdrop on prejudice with neuroimaging and interference studies of the amygdala, we argue that the prevalent ingroup–outgroup interpretation does not fully capture amygdala activity across the race-related social neuroscience literature. Instead, we offer an alternative explanation: that differential amygdala activity may best be considered in terms of threat, arising through culturally-learned associations between black males and potential threat.

## Linking prejudice and the amygdala

**Figure 1:**
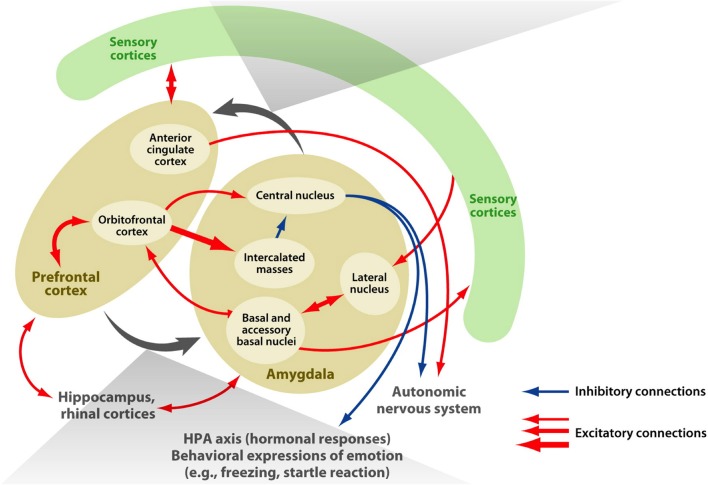
**Connectivity of the amygdala**. The macaque amygdala, comprised of multiple subdivisions, has extensive connections with the frontal lobe. Adapted from Figure 1 (Salzman and Fusi, 2010).

The amygdala is an almond-shaped structure situated in the human medial temporal lobe that is considered important in the acquisition and expression of a range of learned emotional responses (in animals see LeDoux, 1996; for humans see Whalen, 1998; Davis and Whalen, 2001). It comprises several distinct nuclei that receive extensive afferent connections from neocortical areas in all four lobes of the brain, in addition to subcortical thalamic, hippocampal, and cingulate areas. It is critically involved in a myriad of functions including: avoidance conditioning, learned (conditioned) fear, innate (unconditioned) fear, memory for faces, and both positive and negative affect (for reviews see LeDoux, 2007; also Balleine and Killcross, 2006). Mirroring its functional diversity, the structure known as the amygdala in fact encompasses several groups of nuclei with distinct structural and functional characteristics. Independent cytoarchitectonic investigations of post-mortem (human) brains, which allow discrimination according to cell type, have led to general agreement that the amygdala can be subdivided into three major sets of nuclei: the basolateral group (basal, lateral, and accessory basal nuclei), the corticomedial group (cortical and medial nuclei), and the central nucleus (Amaral et al., 1992; de Olmos, 2004; Mai et al., 2008; Nieuwenhuys et al., 2008; Solano-Castiella et al., 2010, 2011). Figure 1 illustrates the subdivisions and connectivity of the macaque amygdala, particularly to frontal lobe regions.

Hart et al.'s (2000) neuroimaging study was the first to examine race-related activity in the amygdala, subsequently found to be positively correlated with indirect measures of racial attitudes (Phelps et al., 2000). Remarkably, however, very few social neuroscience articles have paid due consideration to the complex neuroanatomy of the amygdala: indeed, neither of the two aforementioned seminal reports of race-related modulation of amygdala activity discussed the numerous subdivisions or complex connectivity of the amygdala in any depth (Hart et al., 2000; Phelps et al., 2000).

## The social neuroscientific study of prejudice

While explicit expression of racial prejudice has been declining (Fiske, 1998), implicit prejudice toward social outgroups remains evident on indirect measures (Greenwald et al., 2002). Many explanations for this apparent contradiction have been offered (see Blair, 2001; for a review see Hewstone et al., 2002), but the major factor determining the level of convergence between implicit and explicit measures is believed to be the normative context (Dovidio et al., 1986, 2001). For racial prejudice, it is widely accepted that this divergent trend resulted from socially desirable responding in explicit (self-report) measures of prejudice (Fazio et al., 1995; Hewstone et al., 2002). For instance, social expectations may lead individuals to publically endorse egalitarian values that they do not privately endorse (Devine et al., 2002; Eberhardt, 2005). As such, the majority of modern research now focuses on examining automatic/unconscious racial bias using a combination of techniques. These are often more indirect measures of bias, such as subliminal priming, lexical decision tasks, and implicit association measures, but also more physiologically focused, such as event-related potentials (ERPs), electromyography (EMG), startle eyeblink responses, and functional neuroimaging (Eberhardt, 2005). Indeed, these approaches confer many benefits over traditional explicit measures of bias. Some techniques (fMRI, ERPs) allow investigation of the engagement of different brain regions during interesting behavioral phenomena, offering more sensitive measures of cognitive evaluation than would be available in response time data. Others (e.g., subliminal priming, implicit associations) assess unintentional biases, of which people are largely unaware (Hewstone et al., 2002).

### Amygdala involvement in race-related evaluative processing

In 2000, Hart et al. offered the first fMRI study investigating race-related amygdala activity. It should be noted that the authors directly stated that their research was not aimed at uncovering any racial differences in amygdala activity, rather it was “explicitly designed to assess fMRI responses to outgroup vs. ingroup faces across subjects of both races” (p. 2352). In this study, they presented the faces of black and white people to self-identified black and white participants while recording neural activity in the amygdala. Initially, no significant differences in activity were observed between ingroup and outgroup faces. Subjects then remained in the scanner, but were allowed to rest for a few minutes before a second functional scan. After this short break, participants repeated the experiment, and the authors reported a decrease in amygdala activation in this repeated session that was greater for ingroup faces than outgroup faces, indicating an increased habituation toward ingroup faces. An innocuous explanation exists for this result: greater familiarity to ingroup faces might lead to faster habituation of amygdala responses. Indeed, this repetition suppression-type effect would not be controversial as such effects are common in visual processing, albeit usually on a shorter timescale (see Grill-Spector et al., 2006, for a review). However, Phelps et al. (2000) offered an alternative ingroup–outgroup interpretation: differing amygdala responses could be linked to racial bias against outgroup members.

Phelps et al. (2000, Experiment 1) were the first to examine the link between implicit measures of racial bias and neural activity. During fMRI image acquisition, white participants were exposed to unfamiliar black and white faces while indicating whether successive images were the same as or different from one another. After the scanning session, participants completed both explicit (Modern Racism Scale; McConahay, 1986) and implicit [Implicit Association Test (IAT); Greenwald et al., 1998] measures of racial attitudes. Results showed a significant correlation between differences in amygdala activation and scores on the IAT test, such that the white participants with the most negative implicit attitudes toward blacks exhibited the greatest difference in amygdala activity between responses to black and white faces. In addition, the authors noted a trend (*p* = 0.1) toward a greater startle eyeblink when viewing black compared to white faces when measured approximately 1 week after the fMRI scanning session. Interestingly, this pattern was not observed when famous, well-regarded white and black stimulus faces were used (Phelps et al., 2000, Experiment 2). Taking both of these findings into account, Phelps et al. (2000) interpreted these findings as evidence that amygdala and behavioral responses of white participants to black vs. white faces reflect cultural group-level evaluations modified by individual experience.

Subsequent research has supported, and built upon, these two influential studies. Wheeler and Fiske (2005) offer the most notable example: they had European American participants view photographs of unfamiliar African- and European-American faces and perform a social categorization task, indicating whether the person in each photo was over 21 years old. They reported significantly greater amygdala activity to African American faces compared to European American faces. Cunningham et al. (2004) took an alternative approach: during fMRI recording, white participants were asked in a two alternative forced choice procedure whether a visual stimulus was presented on the left or right of a central fixation cross. Visual stimuli were either black or white faces that were presented either subliminally (30 ms), or for much longer (525 ms). Their observed pattern of results proved illuminating: there was significantly greater amygdala activity only for subliminally presented black vs. white faces. Furthermore, this difference in activity correlated positively with participants' IAT scores such that individuals with more implicit negativity toward blacks relative to whites displayed greater amygdala response to subliminally presented black relative to white faces. Stimulus duration may be one important feature in determining amygdala response to racial faces. When Richeson et al. (2003) presented stimuli for 2 s and asked subjects to report which side of the display the images appeared, they found no significant differences to outgroup vs. ingroup faces in the amygdala BOLD signal, nor did they find significant correlations between IAT scores and amygdala responses.

Other studies demonstrate substantial task-dependence of the amygdala response to racial stimuli. Van Bavel et al. (2008) had participants learn the artificial group membership of 24 facial stimuli and then categorize the faces sequentially according to either group, or racial membership. Here, the authors reported no significant difference in amygdala signal for racial outgroup vs. ingroup, and instead found significantly greater amygdala activity to artificial ingroup faces than outgroup faces, despite there being an equal number of black and white faces in each artificial team. Freeman et al. (2010) compared neural activity when participants made personality judgments about white and black facial stimuli when they were given either irrelevant/non-person-descriptive information (in the superficial judgment condition), or relevant/meaningful information about the faces (individuated judgments). They found superficial judgments recruited the amygdala, whereas individuated judgments recruited a network of regions putatively involved in “mentalizing”/Theory of Mind.

In an interesting subsequent study, Krill and Platek (2009) had participants play *Cyberball*, a three person ball toss game that can be programmed to exclude participants, in a MRI scanner. Although not the primary aim of the study, the authors reported correlations between IAT-D scores [the contrast between the two stimulus blocks (White + Pleasant and Black + Unpleasant) vs. (White + Unpleasant and Black + Pleasant)], and amygdala activity. That is, individuals who showed greater positive bias toward same-race IAT images showed a trend toward increased left and right amygdala activity during other-race game exclusion conditions. However, such results were only uncovered using unilateral amygdala ROIs in separate analyses.

### Summarizing the dominant interpretation

The main things that are consistent amongst the aforementioned studies are: (a) that different modes of encoding race-related information can lead to different patterns of amygdala activation, and (b) an overarching tendency for greater activity in response to black male faces, irrespective of a participant's own race or gender. Indeed, both of these findings are reflected in a recent meta-analysis of ingroup–outgroup social categorization fMRI studies: Shkurko (2013) used an Activation Likelihood Estimation (ALE) analysis to attempt to identify brain structures responsible for social categorization. Shkurko's meta-analysis suggests that simple ingroup–outgroup contrasts are inconsistent: no ALE-based cluster was found for the right amygdala, and the left amygdala cluster was significant for ingroup + outgroup, ingroup, and outgroup contrasts. However, it must be noted that this meta-analysis included any fMRI studies involving social categorization, and therefore included studies using political, age, gender, and minimal group boundaries in addition to the race-related studies considered in the present review.

In summary, prior research findings (Hart et al., 2000; Phelps et al., 2000; Cunningham et al., 2004; Wheeler and Fiske, 2005; Krill and Platek, 2009) have been taken as support for what could be termed an “ingroup–outgroup” interpretation: that the amygdala distinguishes between unfamiliar in-group and out-group faces, with greater activity toward out-groups than in-groups. We believe this interpretation merits further investigation.

### Questioning an ingroup–outgroup interpretation

It might be tempting to interpret these findings as demonstrating that amygdala responses are modulated by race such that activity is greater in response to racial outgroup stimuli than ingroup stimuli. However, Lieberman et al. (2005) found that African American subjects also show greater amygdala activity to African American faces than European American faces. The authors suggest that this result is consistent with evidence that African Americans have negative implicit attitudes toward other African Americans (Nosek et al., 2002). The notion that the amygdala might be categorizing faces in a discrete fashion, such as distinguishing African American and European American faces, can also be questioned. Ronquillo et al. (2007) offer fMRI evidence that race-related amygdala activity is also influenced by skin tone. In this study, white participants judged whether unfamiliar black and white faces with varied skin tone (light, dark) were older or younger than 24 years old. This task design is almost identical to that of Wheeler and Fiske's (2005), and indeed Ronquillo et al. (2007) replicated Wheeler and Fiske's (2005) finding of greater amygdala activity for black vs. white faces. However, this activity was now modulated by skin tone, such that equivalent amygdala activity was elicited by dark or light-skinned black targets, but dark-skinned white targets elicited significantly more activity than light-skinned white targets. Since the researchers did not administer tests of implicit associations, this study is unable to indicate whether the skin-tone modulation of amygdala activity was statistically associated with differences in implicit attitudes toward faces in the different conditions.

In fact, a closer look at the results suggests these studies are far less similar than they might appear. When Phelps et al.'s (2000, Experiment 2) used familiar, positively regarded target stimuli, there was no longer a trend toward eyeblink startle potentiation to black faces, and the imaging data revealed no consistent pattern of amygdala activity when white participants viewed black or white faces. In the Wheeler and Fiske (2005) study, fMRI data were acquired from only seven participants and the significantly higher amygdala activity for black vs. white faces was, only in the left amygdala, across 15 of the 3.75 × 3.75 × 3.75 mm^3^ voxels afforded by their 1.5 T scanner. Furthermore, they observed an entirely different pattern of activation in their second task, requiring participants to decide whether the individual would like a particular vegetable. Here, significant activity was observed across eight voxels in the right amygdala, in the opposite direction such that activity was lower in response to African American faces than European American. In the Cunningham et al. (2004) study [*N* = 13], significant differences in amygdala response to the subliminally presented faces were only observed in an area of the right amygdala which extended substantially into the ventral pallidum. Likewise, Lieberman et al.'s (2005) study requires careful consideration. Once more, amygdala activity was only seen unilaterally, in the right amygdala. Furthermore, when participants verbally, rather than perceptually, encoded the race of target images there was in fact a decrease in amygdala activity for African American compared to European American targets.

In the next section, we offer an alternative interpretation of the social neuroscience literature on race-related amygdala activity. This raises the question of whether the observed pattern of amygdala modulation directly reflects a racial or outgroup bias *per se*, or, as we shall argue, that differential activity stems from perceived threat/uncertainty, which is likely to emerge as a learned bias against black males.

## Amygdala activity as a response to perceived threat

Young black men are often stereotyped as violent, criminal, and dangerous (Trawalter et al., 2009), and research has shown that black men are both implicitly (Payne, 2001; Maner et al., 2005) and explicitly (Cottrell and Neuberg, 2005) associated with threat. Considered in combination with the neuroimaging studies discussed earlier, there is a pervasive connection between black men and threat in the minds of many individuals, irrespective of their own race or gender. As such, we suggest that differential amygdala activity may best be considered in terms of threat, and we correspondingly highlight studies demonstrating bilateral amygdala modulation by threat. More specifically, we then argue that negative culturally-learned associations between black males and potential threat may better explain the data than does a general ingroup–outgroup explanation.

Why might the face of a racial outgroup member be construed as threatening? One possibility, which early research seemed to focus on, is the notion that the mere categorization of a target as “not one of us” activates the “universal outgroup” stereotype, including traits such as dishonest, competitive, and hostile (Campbell, 1967; Richeson et al., 2008). Alternatively, rather than outgroup status itself modulating amygdala activation, it may be the presence of stimulus cues signaling threat, danger, or social importance (Richeson et al., 2008). Indeed, perception of threat has been strongly linked to prejudice in social–psychological accounts of intergroup conflict (Stephan et al., 2002; Jost et al., 2003).

There already exist a priori reasons for investigating the link between the amygdala and threat. Recall that patients with bilateral amygdala damage display deficits in threat detection, overrating the perceived trustworthiness and approachability of strangers compared to healthy participants, due to an inability to use threat-relevant information communicated through the eyes (Adolphs et al., 2005). The eyes are an important source of social information: almost invariably, the most relevant social targets in our surroundings are those who have established direct eye contact (Baron-Cohen, 1995), and emotional events have long been known to evoke pupil dilation (Privitera et al., 2010). In addition to deficits in using threat-relevant information from the eyes, patients with amygdala damage show a general reduction in direct eye contact in social interactions (Spezio et al., 2007). Two important ways in which the eyes change are the direction of gaze, and pupil dilation; manipulations of both features have been shown to cause patterns of differential amygdala activity bilaterally.

### Effect of gaze on amygdala response

It is well documented that biologically threatening stimuli such as snakes and spiders engage processes of selective attention (reviewed by Öhman and Mineka, 2001), but social threats do also (e.g., Fox et al., 2002). One relevant study in this regard by Trawalter et al. (2009) compared response latency in a dot detection task when targets were preceded by subliminally presented black or white faces. Their results showed that white perceivers selectively attend to the faces of young black men, but this effect was eliminated when preceding faces displayed averted eye-gaze (i.e., were less socially relevant). In parallel, early neurophysiological investigations in monkeys by Brothers et al. (1990) identified cells in the medial and lateral nuclei of the amygdala that are sensitive to eye-gaze direction.

Richeson et al. (2008) reported two experiments that directly investigated the effects of eye-gaze and race on neural activity. In Study 1, white participants were presented images of unfamiliar black and white faces with direct or averted eye-gaze for 2.5 s and asked to rate how threatening they were on a 7-point Likert-type scale. Results from this explicit threat rating showed a significant main effect of eye-gaze such that faces with direct-gaze were rated as more threatening than averted-gaze faces, irrespective of race. In Study 2, white participants were required to report whether direct/averted-gaze faces were presented to the left or right of a central fixation cross, under fMRI recording. Results show that black faces with direct gaze elicited significantly greater amygdala activity than black faces with averted gaze. However, the same gaze-modulation of activity was not observed for white faces. Interestingly, overall, black targets only elicited greater amygdala activity than white when the targets displayed direct gaze. For trials when gaze was either averted or eyes were closed, there were no significant differences between amygdala responses to white and black targets. Such results are extremely important, providing substantial evidence against an ingroup–outgroup interpretation. If the ingroup–outgroup explanation were correct, we would expect a main effect of race in the amygdala BOLD signal irrespective of gaze, which was not observed in this study. There was, however, a weak main effect of race in behavioral (Likert scale) ratings of threat. This weaker effect is likely the reason for the absence of a neural difference in the amygdala.

### Unconscious responses to pupil dilation

As mentioned earlier, gaze direction is only one form of socially relevant information conferred by the eyes; pupil dilation is another. Emotional events have long been known to evoke pupil dilation (Privitera et al., 2010). Pupil dilation has been interpreted as a general indicator of heightened vigilance, arousal, and/or interest (Steinhauer et al., 2004; Demos et al., 2008), and thus may be interpreted as another general indicator of threat. Evidence suggests that the amygdala is sensitive to the pupil size of others: Demos et al. (2008) showed participants images of faces whose pupils were either unaltered, or modified to be smaller/larger than their original size. Despite no participant being aware of this manipulation upon debrief, the results show bilateral increases in amygdala activity for faces with relatively large pupils. Indeed, bearing in mind the extensive efferent connections between the amygdala and the sympathetic nervous system, the amygdala is well-placed to signal socially relevant threat signals and instigate fight-or-flight responses. In combination, Richeson et al. (2008) and Demos et al. (2008) demonstrate an important pattern of results: (a) bilateral amygdala modulation is elicited by social markers of threat, compared to inconsistent, unilateral amygdala modulation by racial group membership; and (b) direct eye-gaze can even override the expected basic pattern of race-related activity. Table 1 summarizes the findings of the neuroimaging studies reviewed herein.

**Table 1:** **Reviewing amygdala activity across neuroimaging studies**.

**References**	**Contrast**	**Participants**			**Target stimuli**			**Task**	**Technical details**	
		***N***	**Age**	**Race**	**Expression**	**Gender**	**Race**		**Hemisphere**	**Resolution**
**(A) *UNILATERAL* AMYGDALA MODULATION**										
**(i) No significant effect**										
Hart et al., 2000	Ingroup–Outgroup	8	20–35	B + W	Neutral	M + F	B + W	Male/Female	L	3.13 × 3.13 × 3
Phelps et al., 2000	Black–White^†^	14	–	W	Neutral	M	B + W	Same/Different	n/a	3.13 × 3.13 × 3
Phelps et al., 2000	Black–White (familiar)	13	–	W	Neutral	M	B + W	Same/Different	n/a	3.13 × 3.13 × 3
Cunningham et al., 2004	Black–White^†^	13	27	W	Neutral	–	B + W	Right/Left	n/a	3.13 × 3.13 × 6
Richeson et al., 2003	Black–White	15	21	W	Neutral	–	B + W	Right/Left	n/a	3 × 3 × 3
Richeson et al., 2003	Black–White	15	20	W	Neutral	–	B + W	Right/Left	n/a	3 × 3 × 3
Wheeler and Fiske, 2005	Black–White	7	–	W	Happy	–	B + W	Dot detection	n/a	3.75 × 3.75 × 5
Krill and Platek, 2009	Other-same race^†^	14	28	W	–	–	B + W	Cyberball game	n/a	–
**(ii) Significant *increase***										
Lieberman et al., 2005	Black–White	20	24	B + W	Neutral	M	B + W	Same/Different	R	4 × 4 × 4
Cunningham et al., 2004	Black–White (subliminal)^†^	13	27	W	Neutral	–	B + W	Right/Left	R	3.13 × 3.13 × 6
Wheeler and Fiske, 2005	Black–White	7	–	W	Happy	–	B + W	Age >21?	L	3.75 × 3.75 × 5
Ronquillo et al., 2007	Black–White	11	18–36	W	Neutral	M	B + W	Age >24?	R	4.5 × 4.5 × 3.5
Ronquillo et al., 2007	Dark–Light skin	11	18–36	W	Neutral	M	B + W	Age >24?	R	4.5 × 4.5 × 3.5
**(iii) Significant *decrease***										
Lieberman et al., 2005	Verbal Black–White	21	25	B + W	Neutral	M	B + W	*Verbal* Same/Diff.	R	4 × 4 × 4
Wheeler and Fiske, 2005	Black–White	7	–	W	Happy	–	B + W	Like/Dislike Veg?	R	3.75 × 3.75 × 5
**(B) *BILATERAL* AMYGDALA MODULATION**										
**(i) Significant *increase***										
Richeson et al., 2008	Black–White (direct gaze)	9	19–23	W	Neutral	–	B + W	Right/Left	R + L	3 × 3 × 3
Demos et al., 2008	Dilated-normal pupil	27	22	–	Neutral	F	W	Passive viewing	R + L	3 × 3 × 3
Telzer et al., 2013	B-W correlation with age	32	4–17	M	Varied	–	B + W	Same/Different	R + L	3 × 3 × 3
**(ii) No significant effect**										
Richeson et al., 2008	Black–White (averted gaze)	9	19–23	W	Neutral	–	B + W	Right/Left	n/a	3 × 3 × 3

*Section A highlights inconsistent, unilateral activity across race-related social neuroscience studies. Section B highlights results showing bilateral amygdala activity. Importantly, Target stimuli gender is rarely reported, precluding discussion of differences in activity toward Black men vs. Black women*.

General: “–” for information not disclosed in articles. Contrast: “

† *” indicates reported positive correlation between B-W difference and Implicit Prejudice (IAT). Participants: Range given for age where mean not reported. Technical: Resolution as dimensions of one voxel in mm, compared to the ~1200 mm^3^ volume of the amygdala. All studies conducted in USA*.

### Basic predictions of the threat-based interpretation

This threat-based interpretation makes two important predictions for investigation. Firstly, it suggests that amygdala sensitivity should not occur toward black females, who are not stereotyped in the same way as black males. As seen in Table 1, previous studies rarely indicate the gender of their target stimuli, precluding us from assessing this contrast in the literature. Secondly, participants who do not believe or are not aware of the black male stereotype should not display race-related activity. Devine (1989) showed that participants vary in the extent to which their attitudes reflect features of the black stereotype. By pre-testing participants along this dimension, research could contrast amygdala responses to black and white male face stimuli between participants with and without this stereotype of black males. Here, the classic ingroup–outgroup interpretation would predict greater amygdala response to black faces, whereas the threat-based interpretation predicts no significant difference in amygdala activity.

One further experiment that can help distinguish an ingroup–outgroup vs. a threat-based hypothesis would be to manipulate threat orthogonally to racial group membership, for instance, having neutral vs. angry/overtly threatening black and white faces. In this experiment, we may then expect amygdala sensitivity to be higher for black neutral faces than white neutral, but reduced differences between black and white faces when their expressions are smiling/friendly (and thus threat is eliminated or diffused). The extent to which each participant found each stimulus threatening could then be assessed after the scanning session, using self-report, and then used in analyzing the fMRI data.

A pure test of the ingroup–outgroup interpretation is conferred by minimal group experiments—that is, artificial groups created in the laboratory on the basis of some minimally important criteria, such as a result of a coin toss (Tajfel, 1970). For instance, Van Bavel et al. (2008) assigned participants to mixed-race teams and found greater amygdala BOLD activity when viewing novel ingroup vs. outgroup members, regardless of race. In this instance, groups were arbitrarily assigned, the authors reported no evidence of out-group disliking, and there was no explicit measure of how threatening stimuli were perceived to be. It is difficult to make a threat-based prediction in experiments where it was unlikely that participants found any stimuli to be threatening. In such circumstances, it is highly likely that the amygdala response was being driven by the motivational salience of stimuli (Cunningham and Brosch, 2012).

### Further outgroups

It is clear that not all outgroups evoke threat to the same degree. For instance, black men are likely to be perceived as more threatening than black women, or Asian men or women. Future work could continue to explore the way that the amygdala responds to other outgroups, in particular considering the effect of presenting black female faces to both male and female observers. Since black females do not have the same “threat” associated with them, an experiment that included black, white, male and female faces should determine whether amygdala responses are related to in/out group or threat. Additional insight would be gained if such an experiment included a manipulation of criminality, since a threat-based hypothesis would predict a main effect of perceived criminality on amygdala response. Innovative “reverse correlation” methods offer a powerful tool to extract the fine-grained information of a stimulus underlying its categorization (Mangini and Biederman, 2004; Dotsch et al., 2008, 2011; Dotsch and Todorov, 2011; Todorov et al., 2011a,b) and would enable the independent creation and validation of a set of suitable stimuli, without invoking subjective judgments on the part of the experimenter.

### Boundary conditions

In this review we have proposed that for studies investigating race-related amygdala activity, considering results in terms of threat provides a better fit to the literature than considering results in broader ingroup–outgroup terms. However, that is not to say that threat drives amygdala responses in all circumstances. For instance, while the amygdala is often involved in responses to potentially unsettling stimuli, such as threatening, novel, bizarre, ambiguous, or untrustworthy faces, it has also been found to respond to extremely positive stimuli (Cunningham et al., 2008) and extremely trustworthy faces (Todorov, 2012). Understanding the computational role of the amygdala becomes even more complicated considering other studies reporting non-linear amygdala responses with stronger responses to both negative and positive faces than to faces at the middle of the continuum (Winston et al., 2007; Sergerie et al., 2008; Said et al., 2009; Todorov et al., 2011a,b; Todorov, 2012). However, recalling evidence described earlier demonstrating that the amygdala consists of several subregions with different connectivity patterns, it is plausible that earlier research in the field simply lacked the spatial resolution to discriminate activity in specific amygdala subregions. Given the heterogeneous neuroanatomy of the amygdala, evidence of its engagement in tasks with no manipulation of threat is not evidence against a threat-based interpretation of amygdala engagement in the present review. In such non-threatening conditions, it is more likely that other factors (such as goal-relevance) drive amygdala response. Furthermore, finer-grained analyses of the spatial pattern of amygdala activation might allow investigators to further examine valence-independent activity. In particular, it is important to clarify whether specific sub-populations of neurons are consistently active to positive-valence stimuli and others to negative-valence stimuli, or whether a more general vigilance account is a more appropriate interpretation of amygdala activity. Occasional opportunities for human intracranial recordings in the amygdala might help to address this question. For instance, Rutishauser et al. (2011) recorded from the amygdalae of seven neurosurgical patients and, interestingly, found that only 10.3% of the units they recorded (4/39) were active for fearful vs. happy faces. As such, it is plausible that distinct pools of neurons are valence-selective, but spatially indistinguishable in fMRI studies.

To the extent that threatening stimuli are almost always salient stimuli, we believe that the present review is consistent with a broader motivational salience account of amygdala function (Cunningham and Brosch, 2012; Kubota et al., 2012). However, although motivational salience hypotheses suggest “that the amygdala is involved in processing stimulus relevance for the goals and motivations of the perceiver” (Cunningham and Brosch, 2012, p. 54), patients with amygdala damage do not suffer predominantly deficits of attention; rather, they express a range of broadly threat-related impairments, discussed in section Insights from patient groups of the present review. Importantly, amygdala damage suffered later in life does not eliminate recognition of fearful faces (Adolphs et al., 1999), nor does it eliminate IAT biases (Phelps et al., 2003), suggesting that the amygdala is critical for the acquisition of emotional or threatening associations, rather than their expression/retrieval.

## Future directions

### Modern MRI parcellation

High-resolution MRI could well-afford cleaner inferences about the nature of amygdala activity. Indeed, insufficient spatial resolution to discriminate BOLD activity between subdivisions of the amygdala could explain some of the inconsistencies in social neuroscience literature on prejudice. For instance, it is plausible that different components of social (and racial) interactions, such as threat, ingroup favoritism, or outgroup derogation involve different nuclei within the amygdala. Todorov's (2012) recent review of amygdala involvement in face-perception argued that the population of neurons in the amygdala that are face-selective (typically in the basolateral amygdala) are likely different to those engaging in attentional processes (in the central nucleus). In addition, a recent multi-level kernel density analysis by Mende-Siedlecki et al. (2013) proposes a dorsal/ventral dissociation within the amygdala between populations encoding face intensity and valence, respectively.

### Neurodevelopmental trajectory of race-related amygdala response

One important question remains: how might this pattern of amygdala activity reflecting a perceived threat posed by black men emerge? The proposition that race-related amygdala activity reflects culturally learned messages that African American individuals are potentially threatening has recently received substantial support. Telzer et al. (2013) hypothesized that the differential perception of race associated with amygdala activity is unlikely to reflect innate processes, and instead emerges during development. They used fMRI to investigate the neurodevelopmental trajectory of amygdala response to race across children and adolescents from different racial backgrounds aged between 4 and 16.5 years old. Thirty-two participants (11 African American, 11 European American, 6 Asian American, and 4 Latin American) were shown a trio of emotional faces and had to report which of the two faces at the bottom were expressing the same emotion as the face on the top. For each participant, bilateral amygdala activity to European Americans was subtracted from activity to African Americans and plotted as a function of age.

A positive correlation was found between bilateral amygdala response to African American-European American faces and age, suggesting that the differential activity develops during childhood. Furthermore, the authors showed that this correlation was being driven by the relationship between activity to African American faces and age, since there was no correlation between age and activity to European American faces. Interestingly, the authors examined whether the racial diversity of participants' peers would also modulate the response pattern of the amygdala. They found that greater peer diversity was associated with attenuated (right) amygdala response to African American faces, suggesting that more racially homogenous peer groups relate to greater amygdala response to African American faces. However, once more, without implicit measures of bias we cannot evaluate whether this pattern of race-related amygdala modulation is indicative of more prejudicial beliefs.

In adult human subjects, Bickart et al. (2012) showed a relationship between the size of social networks and strength of connectivity between the amygdalae and cortical areas involved in social perception. As they intimate, this trend toward deconstructing social functions into processes subserved by distinct brain networks would enable future research to better consider relationships between brain function and cognitive phenomena of interest in both healthy and clinical populations. Such an approach may also allow longitudinal measurement of the development of prejudice in children. Moreover, in this instance, research should seek to identify the amygdala networks engaged in differential activation when viewing stimuli varying in threat or racial group membership.

### Effects of reducing implicit bias on amygdala activity

Social psychological research on prejudice has shown that the social-cognitive factors underlying perceiver differences in prejudice are not irreparable, particularly in young children and adolescents (Hewstone et al., 2002). While there are many approaches to bias reduction, many focus on increasing the quantity and quality of intergroup contact. In particular these include: increasing the complexity of social categorizations by highlighting superordinate and dual group memberships (Van Bavel et al., 2008); and reducing the salience of category distinctions by differentiating and personalizing outgroup individuals, and forming common ingroups (see review by Hewstone et al., 2002). Indeed, improved contact/familiarity reducing implicit prejudice converges nicely with evidence discussed earlier that greater peer diversity was linked to attenuated amygdala response to African American individuals (Telzer et al., 2013).

With this in mind, we offer suggestions for future research that would elucidate the nature of the relationship between the amygdala and implicit prejudice. We believe that is feasible to extend the work of Telzer et al. (2013) such that it addresses multiple outstanding questions. Firstly, is race-related amygdala modulation a predictor of implicit prejudice? While we have already discussed evidence in this vein earlier, studies often use less racially-diverse samples (not including Latin, or Asian Americans), and suffer from a lack of statistical power. Addressing these two issues, use of larger and more racially diverse samples would help clarify the relationship in question. This question would also require the addition of IAT measures to the Telzer et al. (2013) study (suitably modified for use on younger participants, or with an older sample). Secondly, is there a parallel developmental trajectory between race-related amygdala modulation and implicit levels of prejudice? If amygdala activity is indeed a predictor of implicit prejudice, one would expect to see a similar positive correlation between increased amygdala response to African American targets and higher levels of implicit prejudice. Finally, and perhaps most interestingly, does reducing implicit prejudice reduce amygdala activity? Research using the IAT has shown that exposure to counter-stereotypic exemplars of a social group decreased bias in both the short- and long-term (Dasgupta and Rivera, 2008), as did asking participants to visualize counter-stereotypical exemplars (Blair et al., 2001). It has also been shown that less prejudiced individuals show less amygdala sensitivity to outgroup faces (Phelps et al., 2000; Cunningham et al., 2004). However, a direct link remains elusive; no study has demonstrated that reducing implicit prejudice reduces race-related amygdala sensitivity. Directly testing this idea would only require a simple within-subjects contrast, achieved by measuring both implicit prejudice and amygdala response to African- and European American faces before and after bias reduction methods such as intergroup contact.

## Insights from patient groups

Occasionally, researchers are presented with patients with very rare patterns of brain damage that can shed light on the function of brain areas. Damage to the amygdala has been shown to impair the ability to recognize social emotions from facial expressions (Adolphs et al., 2005). When compared with healthy participants, patients with bilateral amygdala damage reliably rate the perceived approachability and trustworthiness of strangers higher than do healthy controls (Adolphs et al., 1994). Some 10 years later, the authors further clarified this deficit, suggesting that it results from an inability to utilize threat-relevant information communicated by the eyes of others (Adolphs et al., 2005).

Patient SM046 (Adolphs et al., 1999) suffered from Urbach-Wiethe disease, a case involving bilateral calcification confined to the amygdala. Patient SM046 could not identify the emotion of fear in pictures of human faces, and was unable to draw a fearful face despite identifying and drawing other happy, sad, angry, or disgusted faces (Davis and Whalen, 2001). Interestingly, patients who sustain amygdala damage later in life show normal recognition of fearful faces. Given that patient SM046's damage arose very early in life, it has been suggested that the amygdala is necessary for the acquisition of knowledge about arousing aspects of negative emotions, rather than the retrieval of this knowledge (Adolphs et al., 1999).

A similar pattern of deficits also exists for racial bias. Phelps et al. (2003) reported on Patient SP who had their right amygdala removed as part of a medial temporal lobe resection for intractable epilepsy, in addition to previously observed lesions to their left amygdala. Patient SP still showed an implicit bias in IAT tests, suggesting that the amygdala is not critical for the indirect expression of race bias (Phelps and LeDoux, 2005). In contrast, Santos et al. (2010) reported a lack of racial bias (but not gender bias) in children with Williams Syndrome, a neurodevelopmental genetic disorder in which the amygdala is less active to threatening faces, but shows increased activity to threatening non-social stimuli (Meyer-Lindenberg et al., 2006). Appropriate caution, however, must be taken in any such inferences owing to the lack of specificity in underlying patterns of damage across patients with these conditions. Nonetheless, such examples of amygdala patients expressing deficits in utilizing threat-related information, impaired recognition of fearful faces, and greater approach behavior toward strangers offer an interesting parallel to threat-related hypotheses of amygdala function.

More recently, a group of subjects in South Africa were discovered to share a knock-out-of function mutation of the ECM1 gene resulting in selective bilateral damage to exclusively the basolateral region of the amygdala, leaving other amygdala regions functional and intact (Morgan et al., 2012; Terburg et al., 2012; Van Honk et al., 2013). The van Honk group demonstrate interesting findings: patients with selective basolateral amygdala damage, but intact centromedial amygdala, invest nearly twice as much money in unfamiliar others in a trust game compared to healthy controls (Van Honk et al., 2013). In another study (Morgan et al., 2012) patients with the same condition showed significant working memory (WM) *facilitation* relative to controls, consistent with theories in (WM) research suggesting that WM performance depends on the capacity of the prefrontal cortex to suppress bottom-up arousal-related signals from the amygdala (Postle, 2006). In combination, selective basolateral amygdala patient studies substantiate the argument that different amygdala subdivisions likely subserve different functions.

## Conclusion

In sum, the role of the amygdala in the neural correlates of prejudice has attracted clear interest, but little clarity. Here, we reviewed the social neuroscience literature on race-related amygdala activity against a backdrop of social psychological theories of prejudice and neuroanatomical knowledge of the amygdala. Rather than the dominant interpretation that amygdala activity reflects a racial or outgroup bias *per se*, we argued that this pattern of sensitivity is best considered in terms of potential threat. More specifically, we argued that negative culturally-learned associations between black males and potential threat better explain the observed pattern of amygdala activity than does a wider ingroup/outgroup explanation. While the amygdala is often involved in responses to threat, novel or untrustworthy faces, and ambiguity, this is not to say that all amygdala responses are driven by threat. The amygdala has also been found to respond to extremely positive stimuli (Cunningham et al., 2008) and extremely trustworthy faces (Todorov, 2012). In such non-threatening conditions, it is likely that other factors (such as goal-relevance) drive amygdala response.

Combining state-of-the art neuroimaging in moderate population sizes with implicit behavioral measures of bias could provide conclusive evidence to support these assertions and we have offered specific experiments and predictions to this end.

## Author contributions

Adam M. Chekroud and Miles Hewstone designed the review. Adam M. Chekroud wrote the paper. Adam M. Chekroud, Jim A. C. Everett, Holly Bridge, and Miles Hewstone commented on and edited the paper at all stages.

### Conflict of interest statement

The authors declare that the research was conducted in the absence of any commercial or financial relationships that could be construed as a potential conflict of interest.
